# Real-Time EEG Signal Enhancement Using Canonical Correlation Analysis and Gaussian Mixture Clustering

**DOI:** 10.1155/2018/5081258

**Published:** 2018-01-15

**Authors:** Chin-Teng Lin, Chih-Sheng Huang, Wen-Yu Yang, Avinash Kumar Singh, Chun-Hsiang Chuang, Yu-Kai Wang

**Affiliations:** ^1^School of Software, Faculty of Engineering and Information Technology, University of Technology Sydney, Ultimo, NSW, Australia; ^2^Brain Research Center, National Chiao Tung University, Hsinchu, Taiwan; ^3^College of Biological Science and Technology, National Chiao Tung University, Hsinchu, Taiwan

## Abstract

Electroencephalogram (EEG) signals are usually contaminated with various artifacts, such as signal associated with muscle activity, eye movement, and body motion, which have a noncerebral origin. The amplitude of such artifacts is larger than that of the electrical activity of the brain, so they mask the cortical signals of interest, resulting in biased analysis and interpretation. Several blind source separation methods have been developed to remove artifacts from the EEG recordings. However, the iterative process for measuring separation within multichannel recordings is computationally intractable. Moreover, manually excluding the artifact components requires a time-consuming offline process. This work proposes a real-time artifact removal algorithm that is based on canonical correlation analysis (CCA), feature extraction, and the Gaussian mixture model (GMM) to improve the quality of EEG signals. The CCA was used to decompose EEG signals into components followed by feature extraction to extract representative features and GMM to cluster these features into groups to recognize and remove artifacts. The feasibility of the proposed algorithm was demonstrated by effectively removing artifacts caused by blinks, head/body movement, and chewing from EEG recordings while preserving the temporal and spectral characteristics of the signals that are important to cognitive research.

## 1. Introduction

Electroencephalography (EEG), which is the most convenient brain imaging tool that reveals electrical activity in the brain, has the most near-term potential for real-time applications in everyday environments [1]. However, EEG signals are contaminated with various artifacts, such as signal associated with muscle activity, eye movement, and body motion, which are not of cerebral origin. These artifacts appear as high-amplitude or high-frequency bursts of activity and may be confused with abnormalities, leading to misinterpretation [2]. Numerous signal-preprocessing methods have been proposed for eliminating artifacts that are generated by eye movements or muscle activity [3–10]. Low- and high-pass filters are commonly utilized to remove muscle artifacts and drift artifacts, respectively. However, the spectral patterns of artifacts usually overlap those of brain signals of interest. Frequency filters not only remove artifacts but also suppress informative brain signatures [10]. Recently, advanced machine learning methods [3–10] were developed to deal with artifacts. Most of these methods have three main parts which are source separation, feature extraction, and classification. Concerning source separation, independent component analysis (ICA) [11–13], which yields maximally temporally independent signals from the EEG recording, is a powerful tool for separating brain activity from artifacts. However, the iterative process for measuring independence from EEG signals within multichannel recordings is computationally intractable [14]. Additionally, manually excluding the ICA components that are associated with artifacts is challenging and time-consuming.

Canonical correlation analysis (CCA) has been demonstrated to outperform ICA and frequency filters in eliminating muscle artifacts (electromyography, EMG) [8, 10, 15]. The CCA-based method exploits the fact that the autocorrelation of muscle activity is weaker than that of brain activity. The use of a correlation threshold enables the CCA to identify muscle activity automatically. The objective function of CCA has a closed-form solution that facilitates the real-time implementation of CCA for removing artifacts.

An efficient method for identifying artifacts is informative features, the frequency, spatial, and temporal domains of EEG signals [6]. These features are used jointly rather than independently. For example, spectral powers are not efficient for identifying ocular artifacts, because EEG signals and ocular activity exhibit similar spectral properties around the frequency band of brain activity of interest but temporal features, such as fractal dimension [16] and higher-order statistics [17], are more useful for identifying ocular artifacts.

Feature extraction is often followed by classification. Most classification systems for artifact removal use a supervised learning method [18, 19] that requires labeled data as input for training model. The process of distinguishing artifacts from nonartifacts by visual inspection can be time-consuming, and the result is based on previous experience of the inspector. Therefore, an effective method for labeling artifacts and nonartifacts automatically should be developed. This work proposes a real-time artifact removal algorithm that performs three main tasks—source separation using CCA, artifact feature extraction using spectral and temporal features, and classification based on Gaussian mixture model. To evaluate the performance of the proposed method, two standard visual-evoked tasks, a visual-evoked potential (VEP) task and a steady-state visually evoked potential (SSVEP) task, are performed.

## 2. Materials and M**eth**ods

### 2.1. Participants and Experiment

**Figure 1:**
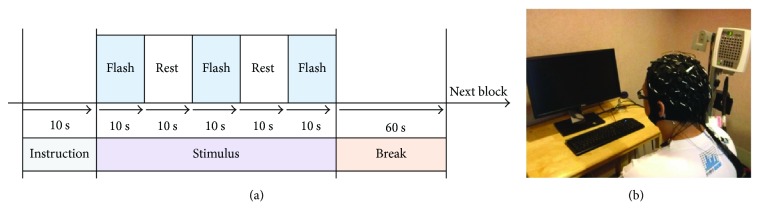
(a) Experimental paradigm. (b) Experimental setup.

Eleven right-handed adults (seven males and four females, aged from 18 to 27 years) were recruited to participate in the study. None of the participants had a history of psychological disorders. Following a detailed explanation of the experimental procedure, all participants completed a consent form before participating. All subjects were required to wear a wired EEG cap with 62 Ag/AgCl electrodes, including 60 EEG electrodes and two reference electrodes (opposite lateral mastoids) (Figure 1(b)). The placement of the EEG electrodes was consistent with the modified international 10–20 system. The contact impedance between all electrodes and cortex was maintained below 5 kΩ. The EEG recordings were collected using a Scan SynAmps2 Express system (Compumedics Ltd., VIC, Australia). The EEG recordings were digitally sampled at 1 kHz with a 16-bit resolution. EEG signals were referenced to the opposite lateral mastoids.

All subjects were instructed to look at the center of a screen and follow the audible instructions to generate artifacts of various types purposefully. Subjects participated in four experimental runs; in one, no motion was performed, and in the other three, common artifacts (blinking, chewing, and head rotation) were generated. Each run involved two sessions. Each session comprised three parts, which were instruction, stimulation, and resting (Figure 1(a)). In the instruction period, the speaker instructed the subjects to perform a particular motion when the flicker stimulus was presented. To prevent habituation, in the stimulus period, the flicker stimulus was presented for 10 s, and the subjects rested for 10 s after that (Figure 1(a)). A 60 s resting break after each session enabled the eye muscles to relax. The screen monitor (DELL U2311Hb, 23^″^, 60 Hz, 1920 × 1080) was used to present the visual stimuli. The distance between the subject's head and the screen was 30 cm (Figure 1(b)).

The flicker stimulus experiment was conducted in a shielded room to prevent any unwanted artifact from appearing in the EEG data. The two flickering stimuli had frequencies of 1 Hz and 15 Hz to induce visual-evoked potential (VEP) and steady-state visual-evoked potential (SSVEP), respectively.

### 2.2. Proposed Artifact Removal Algorithm

**Figure 2:**
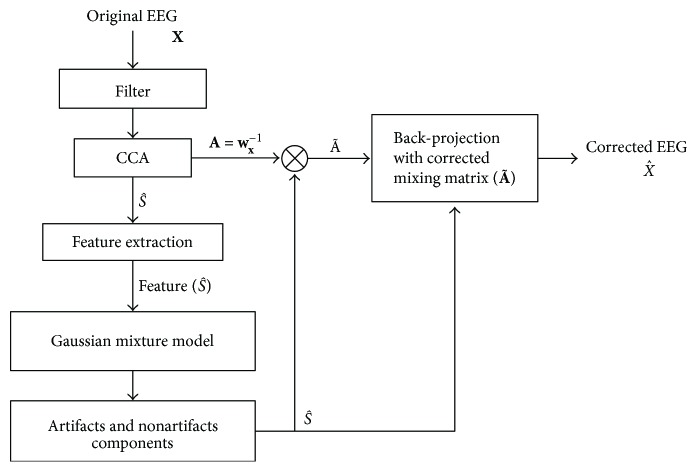
Flowchart of proposed artifact removal algorithm.

The proposed algorithm comprises three main parts, which are CCA, artifact feature extraction, and the Gaussian mixture model after preprocessing, which involves downsampling to 256 Hz and passband filtering of 0.1–60 Hz.  Figure 2 shows the flowchart of proposed artifact removal algorithm.

#### 2.2.1. Canonical Correlation Analysis and EEG Reconstruction

Let the observed EEG signals be **X**(*t*) = [**x**_1_(*t*), **x**_2_(*t*),…,**x**_*M*_(*t*)]^*T*^, *t* = 1, 2,…, *N*, where *N* is the number of samples and *M* represents the number of EEG electrodes used to make the observations. The purpose of blind source separation (BSS) is performed to recover the sources *S*(*t*) = [*s*_1_(*t*), *s*_2_(*t*),…,*s*_*M*_(*t*)]^*T*^ from only sensor observations **X**(*t*). In BSS, **X**(*t*) = [**x**_1_(*t*), **x**_2_(*t*),…,**x**_*M*_(*t*)]^*T*^ is the mixture of a set of unknown source signals *S*(*t*), which is regarded as a linear combination,

(1) $$\begin{array}{c} X\left(t\right)=A\cdot S\left(t\right), \end{array}$$

where **A** is the unknown mixing matrix. The unknown source signals *S*(*t*) are derived by introducing the demixing matrix **W**,

(2) $$\begin{array}{c} \hat{S}\left(t\right)=WX\left(t\right), \end{array}$$

where $\hat{S}\left(t\right)$ approximates the unknown source signals in *S*(*t*), and ideally, **W** is the inverse of the unknown mixing matrix **A**.

Canonical correlation analysis (CCA) is one of the BSSs, which solves the problem by forcing the sources to be maximally autocorrelated and mutually uncorrelated; it has been extensively used to separate muscle artifacts from other EEG activity [8, 10]. In CCA, **Y**(*t*) is the instantly delayed version of the observed EEG signals **X**(*t*) such that **Y**(*t*) = **X**(*t* − 1), and the mean of each row of the matrix **X**(*t*) and **Y**(*t*) is removed to derive two sets of basis vectors for **X** and **Y**. Suppose two canonical variables, *U* and *V*, are linear combinations of the components in **X** and **Y**,

(3) $$\begin{array}{c} \begin{array}{l} U\left(t\right)=w_{x}^{T}X\left(t\right),\\ V\left(t\right)=w_{y}^{T}Y\left(t\right). \end{array} \end{array}$$

CCA is used to find the matrices **w**_**x**_ = [*w*_*x*_1__ ⋯ *w*_*x*_*M*__] and $w_{y}\left[\begin{array}{ccc} w_{y_{1}} & \cdots & w_{y_{M}} \end{array}\right]$ that maximize the correlation *ρ* between *U* and *V*; the following problem has to be solved:

(4) $$\begin{array}{c} \underset{W_{X},W_{Y}}{\max}\rho \left(U,V\right)=\frac{w_{x}^{T}C_{xy}w_{y}}{\sqrt{\left(w_{x}^{T}C_{xx}w_{x}\right)\left(w_{y}^{T}C_{yy}w_{y}\right)}}, \end{array}$$

where **C**_**xx**_ and **C**_**yy**_ are the autocovariance matrices of **X** and **Y**, and **C**_**xy**_ = **C**_**yx**_^*T*^ are the cross-covariance matrices of **X** and **Y**. This optimization problem can be solved by solving the partial derivative with respect to **w**_**x**_ and **w**_**y**_, respectively, and transforming it into the following eigenvalue problem:

(5) $$\begin{array}{c} \begin{array}{c} C_{xx}^{-1}C_{xy}C_{yy}^{-1}C_{yx}w_{x}=\rho^{2}w_{x},\\ C_{yy}^{-1}C_{yx}C_{xx}^{-1}C_{xy}w_{y}=\rho^{2}w_{y}, \end{array} \end{array}$$

where **w**_**x**_ and **w**_**y**_ are eigenvectors, and the canonical autocorrelation coefficient *ρ*^2^ ∈ [0  1] is the eigenvalue. (*u*_*i*_(*t*), *v*_*i*_(*t*)) represents the *ith* pair of canonical variates, and *ρ*_*i*_ is the correlation between *u*_*i*_(*t*) and *v*_*i*_(*t*). In CCA, *U*(*t*) is related to the derived components $\hat{S}\left(t\right)$ as follows:

(6) $$\begin{array}{c} \hat{S}\left(t\right)=WX\left(t\right)=U\left(t\right)=w_{x}^{T}X\left(t\right). \end{array}$$

The unknown mixing matrix **A** can be obtained from the inverse of the demixing matrix **W** = **w**_**x**_: **A** = **w**_**x**_^−1^.

The corrected EEG signals $\hat{X}\left(t\right)$ can be derived from the back-projection of derived components,

(7) $$\begin{array}{c} \hat{X}\left(t\right)=\tilde{A}\cdot \hat{S}\left(t\right), \end{array}$$

where $\tilde{A}$ is the corrected mixing matrix, whose columns, which represent artifact components, contain elements that are set to zero.

**Figure 3:**
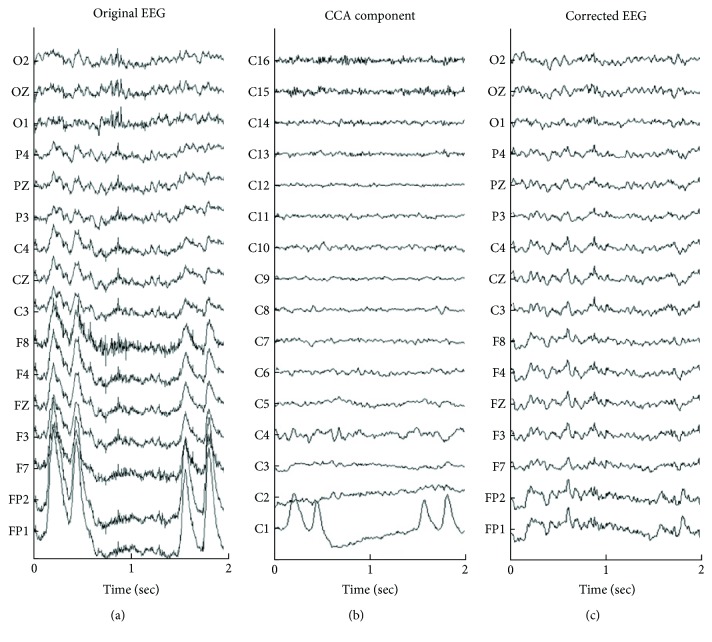
Demonstration of removal of EEG artifacts by BSS-CCA. (a) A 2 s portion of EEG time series that contains blinking. (b) Corresponding CCA component activations. (c) EEG corrected by removing C1, C2, C15, and C16 from (b).

Figure 3(a) shows a 2 s portion of the recorded EEG time series selected from 16 EEG electrodes on scalps, and Figure 3(b) presents the derived CCA component activations, with the order of CCA components sorted (from largest to smallest) by the autocorrelation coefficient. The blink artifact was isolated to as C1; the slow artifact was isolated as C2, and the muscle artifacts were isolated as C15 and C16.  Figure 3(c) shows the artifact-free corrected EEG signals that were obtained by removing four selected components (C1, C2, C15, and C16). Although the CCA was initially proposed to eliminate muscle artifacts, it also successfully separates out slow artifacts.

#### 2.2.2. Feature Extraction

Different types of artifact in EEG typically have different characteristics. For example, the amplitudes of ocular or body movement artifacts are usually much higher than those of the EEG activities of interest. High-frequency and low-amplitude activities accompany muscle artifacts. Therefore, this work proposes ten features—six spectral and four temporal—to reflect the variability of CCA components.

Six spectral features are extracted from the power spectral density (PSD) by fast Fourier transform (FFT) into six specific frequency bands (lower *δ* (0.5~2 Hz), higher *δ* (0.5~4 Hz), *θ* (4~8 Hz), *α* (8~12 Hz), *β* (13~30 Hz), and low *γ* (30~60 Hz)). Four temporal features are extracted from the canonical autocorrelation coefficient, kurtosis, skewness, and fractal dimension (FD), respectively. The canonical autocorrelation coefficient was obtained by CCA. The separated muscle artifact components have the lowest autocorrelation coefficient, and the slow artifact components have the highest autocorrelation coefficient. Kurtosis here is the degree of the peaked distributions of EEG signal and is used to compare degrees of non-Gaussianity of random variables [6, 20]. If the component waveform is highly gathered around the central distribution value, then, the kurtosis is positive. Skewness measures the degree of asymmetry of distribution [6, 20]. If the distribution of the EEG signal of the component waveform is symmetrical, then, the skewness is zero. Ocular or body movement-induced components may exhibit an asymmetrical distribution. The fractal dimension (FD) can quantify the complexity of a waveform, and it is extensively used in the detection of ocular artifacts [21]. In this work, the FD is obtained using Sevcik's algorithm, which is quite robust and allows for fast computation. Sevcik's algorithm firstly maps *n*-point waveform, which has coordinates (*x*_*i*_, *y*_*i*_),  *i* = 1,…, *n*, into a unit square. Let the normalized abscissa of the square be *x*_*i*_^∗^, and the normalized ordinate of the square be *y*_*i*_^∗^, which can be defined as

(8) $$\begin{array}{c} \begin{array}{l} x_{i}^{*}=\frac{x_{i}}{x_{\max}},\\ y_{i}^{*}=\frac{y_{i}-y_{\min}}{y_{\max}-y_{\min}}, \end{array} \end{array}$$

where *x*_max_ is the maximum of *x*, and *y*_max_ and *y*_min_ are the maximum and minimum of *y*, respectively. The FD of the waveform is approximated as

(9) $$\begin{array}{c} FD=1+\frac{\ln\left(l\right)}{\ln\left(2\left(n-1\right)\right)}, \end{array}$$

where *l* denotes the total length of the waveform.

#### 2.2.3. Gaussian Mixture Model

The Gaussian mixture model (GMM) is used for unsupervised learning. It is a probabilistic model that assumes that all data points are generated from a mixture of a finite number of Gaussian distributions with unknown parameters [22]. The GMM consists of *K* Gaussian components as

(10) $$\begin{array}{c} P\left(x\left|\mu ,\Sigma \right.\right)=\overset{K}{\underset{i=1}{\sum }}g\left(x\left|\mu_{i},\Sigma_{i}\right.\right), \end{array}$$

with mean vector **μ**_**i**_ and covariance matrix **Σ**_**i**_. Each probability density of GMM component has the form

(11) $$\begin{array}{c} g\left(x\left|\mu_{i},\Sigma_{i}\right.\right)=\frac{1}{\sqrt{{\left(2\pi \right)}^{d}\left|\Sigma_{i}\right|}}\exp\left\{-\frac{1}{2}{\left(x-\mu_{i}\right)}^{T}\Sigma_{i}^{-1}\left(x-\mu_{i}\right)\right\}. \end{array}$$

Consider *n* samples, with the following likelihood function of GMM:

(12) $$\begin{array}{c} P\left(X\left|\mu ,\Sigma \right.\right)=\overset{n}{\underset{i=1}{\prod }}P\left(x_{i}\left|\mu ,\Sigma \right.\right). \end{array}$$

The parameters are estimated from the maximum likelihood function. The parameters **μ** and **Σ** are obtained by maximizing the likelihood function of GMM (*P*(*X* | **μΣ**)). The GMM function implements the expectation-maximization (EM) algorithm for fitting mixed Gaussian models.

### 2.3. Data Processing

In this work, the continuous EEG signals are processed using a fixed length window (2 s) with an overlapping window (1.5 s). Therefore, the 50 s of raw EEG data in each stimulus block were segmented into 117 2 s epochs, yielding a total of 936 epochs for each subject. The EEG signals for each epoch were decomposed into components by CCA. Traditionally, a neurophysiologist manually labels all data as ocular artifacts, EMG components, or EEG components of interest by inspecting the time series, the power spectral density, or topography of the components. The EEG dataset contains tens of thousands of individual EEG patterns from and across individual participants. In this work, 11 × 936 × 50 = 514,800 (number of subjects × number of epochs × number of EEG channels) CCA components are involved. Manually labeling various types of artifact among these decomposed CCA components is difficult and time-consuming. Therefore, GMM clusters them automatically to identify various extracted features. Typically, researchers/experts manually score and label many common artifacts, such as those associated with blinking, muscle activity, and motion, and the EEG signals of interest by checking the temporal and spectral properties of decomposed components in each GMM cluster. In this work, the Fisher criterion tr(*S*_*w*_^−1^*S*_*b*_) [23] was used to assess the number of GMM clusters; *S*_*b*_ and *S*_*w*_ are the class between and within scatter matrices, and tr(**A**) represents the trace of the square matrix **A**. A larger tr(*S*_*w*_^−1^*S*_*b*_) implies a larger separability in feature space, so the number of GMM clusters can be obtained by finding the largest value of the Fisher criterion variable.

### 2.4. Performance Evaluation

Performance of algorithm has been compared with the most popular method of artifact removal called artifact subspace reconstruction (ASR) [24, 25]. ASR is the most common and widespread online method of removing transient, high-amplitude-related artifacts from different sources like eye blinks, muscle bursts, and movement [26] while recovering essential EEG background activities that lie in the subspace spanned. ASR method is available with EEGLAB [27], and comparison has been performed with nondefault parameters of a sliding window (500 ms), a threshold of three standard deviations, and without any channel rejection.

## 3. Experimental Results

### 3.1. Number of Clusters of GMM and Extracted Features

**Figure 4:**
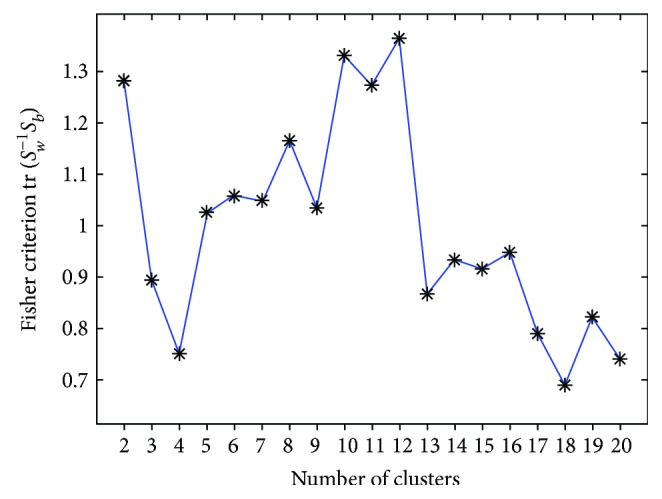
Fluctuation of Fisher criterion value from two to 20 clusters.

In this work, the number of the cluster was obtained by finding the maximum value of the Fisher criterion variable over a set (2, 3,…,20).  Figure 4 shows the estimated values of the Fisher criterion as the number of clusters was varied from two to 20. The number of GMM clusters was set to 12, based on a grid search.

**Figure 5:**
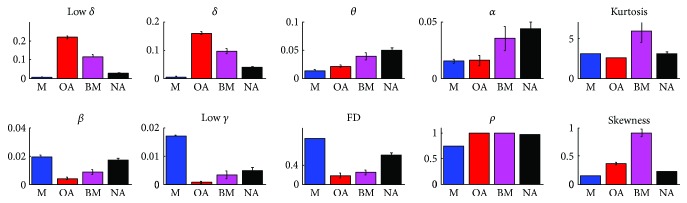
Histogram of artifact and nonartifact significance for extracted features. M, OA, BM, and NA represent muscle artifact, ocular artifact, body movement artifact, and nonartifact activity.

In the experiment design, various typical artifacts, which were blinking, chewing, and head rotation, were generated following the instructions during the experiment. GMM clusters were grouped into four classes, which corresponded to muscle artifacts, ocular artifacts, body movement artifacts, and nonartifacts, by visually inspecting the temporal waveforms and PSDs of EEG signals.  Figure 5 presents the feature histograms for four classes, and most of the classes differed significantly (*p* < 0.001, Wilcoxon signed rank test) between any two classes, with the following exceptional pairs. The first pair was *α*-power features between the muscle activity class and the ocular artifacts class and between the body movement class and the nonartifact activity class. The second pair was the low *γ*-power features between the body movement class and the nonartifact activity class, and the third pair was the FD feature between the ocular artifacts and the body movement, and the kurtosis feature was between the muscle activity class and the nonartifact activity class.

### 3.2. EEG Data

**Figure 6:**
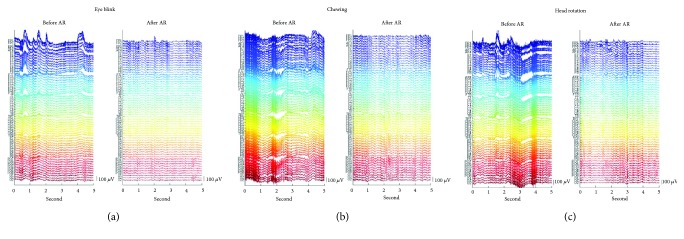
Five seconds of EEG data from a representative subject with (a) blinking, (b) chewing, and (c) head rotation. In each subfigure, left and right plots are obtained before and after the proposed artifact removal algorithm is applied.

Three 5 s long EEG data were selected to illustrate the effectiveness of the proposed artifact removal algorithm (Figure 6). Under all conditions, the amplitude of EEG signals before artifact removal was larger than that after artifact removal. For chewing and head rotation, the EEG signals were contaminated with not only muscle artifacts (Figures 6(b) and 6(c)) but also slow fluctuations.  Figure 6(a) shows EEG signals recorded over the prefrontal and frontal area, and Figures 6(b) and 6(c) show EEG signal recorded over all channels. Exclusion of the CCA components made of slow fluctuations and muscle activity was visible in EEG signals using the proposed artifact removal algorithm.

**Figure 7:**
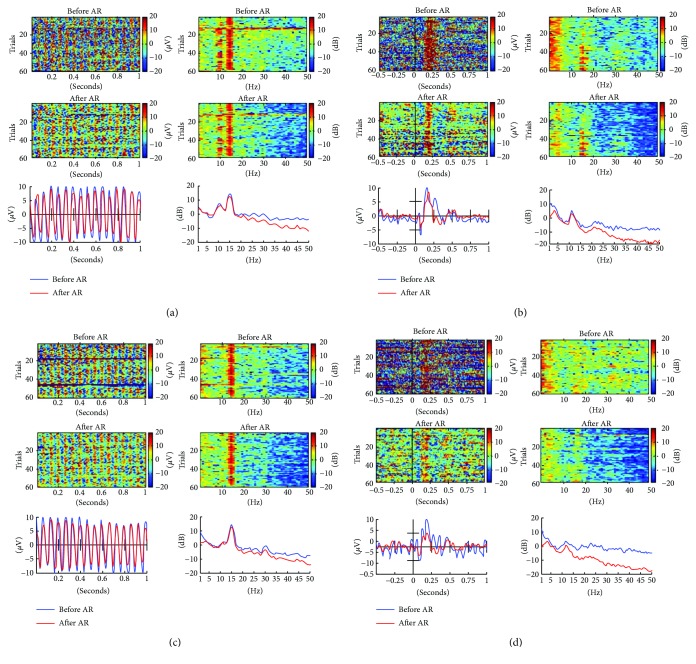
Temporal waveform and power spectrum activities of Oz-EEG channel from a representative subject. (a and c) SSVEP task. (b and d) VEP task. (a and b) Chewing. (c and d) Head rotation. Left and right-hand images in subfigures show EEG waveforms and corresponding log power activity, respectively. Top and middle 2D images show EEG results before and after artifact removal, respectively, and bottom images present averaged results. Each horizontal line in 2D images represents a single trial. The time zero on the *x*-axis represents the time of onset of the visual stimulus. Blue and red lines in bottom images show averaged results obtained before and after artifact removal, respectively. *Note*: AR indicates artifact removal.

**Figure 8:**
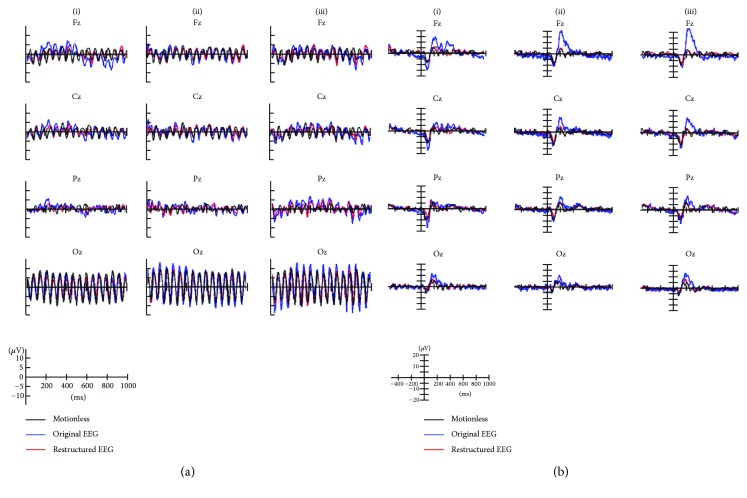
Averaged evoked potentials in Fz, Cz, Pz, and Oz channels during (a) SSVEP and (b) VEP tasks from a representative subject. Each subplot figure shows the averaged waveforms for three artifacts: (i) blinking artifacts, (ii) head rotation artifacts, and (iii) chewing artifacts. In each panel, black line presents averaged waveform without motion, whereas blue and red lines present averaged waveforms before and after artifact removal, respectively.

Temporal (ERPs) and spectral (PSDs) responses of EEG in VEP and SSVEP tasks were used to evaluate the effectiveness of the proposed artifact removal algorithm. In the VEP task, EEG epochs were extracted from 500 ms before to 1000 ms after the visual stimulus onset. In the SSVEP task, EEG epochs were extracted from 0 ms to 1000 ms after the beginning of the visual stimulus onset. The power spectrum activities of the EEG signals were calculated by fast Fourier transformation (FFT) and converted to decibels by taking their log power. The EEG waveforms and the power spectrum activities were vertically stacked by epochs, yielding 2D images (Figure 7), and the averaged ERPs and power spectrum activities from all epochs are shown in Figures 7 and 8.

**Figure 9:**
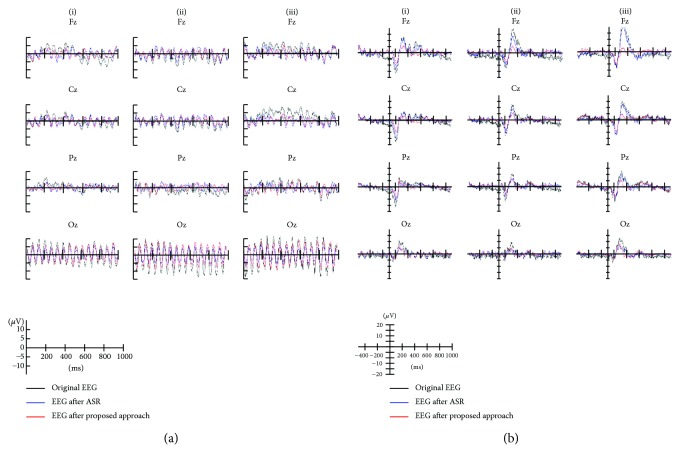
Averaged evoked potentials in Fz, Cz, Pz, and Oz channels during (a) SSVEP and (b) VEP tasks from a representative subject. Each subplot figure shows the averaged waveforms for three artifacts: (i) blinking artifacts, (ii) head rotation artifacts, and (iii) chewing artifacts. In each panel, black line presents averaged waveform without motion, whereas blue and red lines present averaged waveforms after ASR and after proposed artifact removal method, respectively.

Visual stimuli accompany visual-evoked potentials (VEPs) at most recording scalp sites, including the occipital, parietal, central, and frontal electrode sites [28]. Therefore, only results from EEG channels (Fz, Cz, Pz, and Oz) are shown. The proposed artifact removal algorithm reduced the EEG spectral power at high frequency (>20 Hz) during chewing and head rotation (Figure 7). Under all artifact-generating conditions, before artifact removal, the amplitudes and waveforms of ERPs did not appear similar to those under motionless conditions; following artifact removal, the ERPs were similar to those under motionless conditions (Figure 8). Comparison with ASR has been made which shows (Figure 9) that the proposed artifact removal algorithm not only eliminates the influence of artifacts on EEG signals but also maintains the specific frequency response and the temporal profiles in the defined task (Figure 9(a)).

## 4. Discussion

The performance of the proposed artifact removal algorithm was evaluated by classical visual-evoked tasks with common artifacts. Results thus obtained demonstrated that common artifacts in EEG were successfully suppressed by the proposed artifact removal algorithm. EEG signals appeared much cleaner after artifact removal than before it (Figure 6). Moreover, temporal and spectral properties of EEG signals in visual-evoked tasks were maintained after the proposed algorithm was applied and EEG signals had similar profiles to those under motionless condition. The proposed algorithm effectively eliminated artifacts from EEG signals while retaining the featured related properties of EEG activity.

One widely used BSS method in EEG studies is independent component analysis (ICA), which decomposes EEG signals into a set of statically independent components by maximizing its statistical independence. Several methods of ICA-based artifact removal have been proposed for removing artifacts from contaminated EEG signals [6, 9, 20, 29, 30]. Although ICA method can clearly capture the artifacts from EEG signals and separate them from the components of brain activity, it may not be ideal for detecting muscle-related components [31]. ICA requires an iterative process to solve the optimization problem of maximizing the statistical independence among components, which requires extensive computational resources and time. Therefore, the ICA-based artifact removal algorithm cannot be used in real-time applications. Recent studies [8, 10, 15] have demonstrated that CCA outperforms ICA and the traditional filter approach to solve the muscle artifact problem. A CCA-based online approach for artifact removal [8] has also been proposed for removing muscle artifacts from EEG signals. However, these CCA-based methods do not recognize or remove slow artifacts, such as eye movements including blinking. Based on our observations of CCA components derived from CCA-method, CCA can completely separate slow artifacts from brain signals (C1 and C2 in Figure 3(b)); in particular, slow artifacts have higher autocorrelation coefficients and comparable amplitudes than brain signals. Therefore, this work also uses CCA for the real-time separation of brain and artifact activities.

Standard methods involve specific feature extraction and learning classification. Feature extraction is a highly efficient means of achieving satisfactory artifact classification performance. If extracted features can achieve high separability, then, it is easier to distinguish between different classes. Several studies [6, 10, 20, 21, 32] have demonstrated that spectral activities, the canonical autocorrelation coefficient, kurtosis, skewness, and fractal dimension are useful indices for identifying visual, cardiac, or muscle artifacts. This work uses these features for identifying artifact and presents evidence that the extracted features are effective in distinguishing between artifacts or between nonartifact components. Most artifact learning classification requires supervised learning, which requires labeled data to be input to a training model [6, 18, 19]. Although manual labeling can identify artifact and nonartifact component activities, the selection of artifactual components is subjective and needs to be carried out by an expert in the field [33]. In fact, the EEG dataset contains tens of thousands of individual EEG patterns of every participant. This work involves recorded data with 514,800 CCA components; for such a large EEG dataset, manual scoring is impractical, time-consuming, and inconvenient. Unsupervised learning methods are considered in artifact component classification. This work considers GMM because it utilizes well-studied statistical inference techniques and provides flexibility in the mixing of distributions and overall density for each cluster by the mixture model [34]. The number of clusters was obtained by finding the maximum value of the Fisher criterion variable, which provides the highest separability in distinguishing among clusters. Based on the results of the GMM, typical artifacts and EEG activities of interest for all clusters are examined. Typical artifacts (such as those associated with eye movement, muscle activity, and other motion) should be scored for assessment by checking the overall properties of CCA component in GMM cluster.

This work evaluated the efficacy of the proposed artifact removal algorithm for EEG signals for the activities that are likely to produce artifacts during the experiment. Despite its contributions, this work has certain limitations. The collected data are limited to typical artifacts (ocular, muscle, and body movement artifacts). Unlike in laboratory settings, many unknown and typical application-specific artifacts may be generated in real-world situations. For example, an EEG experiment involving car driving on a real road, a speed breaker, or emergency braking may produce atypical artifacts in the EEG recording. If extracerebral artifacts make informative cortical-generated signals very noisy, then, the presented visual-evoked phenomenon in EEG signals does not exist; therefore, no such components were separated and recovered. Finally, the proposed algorithm was applied to EEG signals in two typical visual-evoked tasks. Other tasks should be carried out to evaluate the efficacy of the proposed artifact removal algorithm. Efforts are underway to examine these problems.

## 5. Conclusion

This work proposes a real-time EEG artifact removal algorithm, involving CCA, artifact feature extraction, and the GMM. The efficacy of the proposed method was demonstrated using two classical visual-evoked tasks, which were regular screen flashes with frequencies of 1 Hz and 15 Hz and user-generated artifacts. This work proposes the GMM-based methodology to cluster all components automatically and intelligently to reduce the inconvenience and complexity of labeling the training dataset manually. Experimental results show the feasibility of using the proposed approach to remove artifacts from EEG signals while retaining the properties of *visual-evoked potential information*. In this work, the GMM is projecting component in the labeled dataset which may cause artifact components to be wrongly classified in CCA. Efforts are underway to use transfer learning to classify artifact components more accurately.
